# Elucidating the interaction between stretch and stiffness using an agent-based spring network model of progressive pulmonary fibrosis

**DOI:** 10.3389/fnetp.2024.1396383

**Published:** 2024-05-22

**Authors:** Joseph K. Hall, Jason H. T. Bates, Ramaswamy Krishnan, Jae Hun Kim, Yuqing Deng, Kenneth R. Lutchen, Béla Suki

**Affiliations:** ^1^ Department of Biomedical Engineering, Boston University, Boston, MA, United States; ^2^ Department of Medicine, University of Vermont, Burlington, VT, United States; ^3^ Center for Vascular Biology Research, Department of Emergency Medicine, Beth Israel Deaconess Medical Center and Harvard Medical School, Boston, MA, United States; ^4^ Department of Mechanical Engineering, Boston University, Boston, MA, United States

**Keywords:** mechanotransduction, mechanosensitive, extracellar matrix, fibroblasts, network effects, bifurcation (mathematics)

## Abstract

Pulmonary fibrosis is a deadly disease that involves the dysregulation of fibroblasts and myofibroblasts, which are mechanosensitive. Previous computational models have succeeded in modeling stiffness-mediated fibroblasts behaviors; however, these models have neglected to consider stretch-mediated behaviors, especially stretch-sensitive channels and the stretch-mediated release of latent TGF-β. Here, we develop and explore an agent-based model and spring network model hybrid that is capable of recapitulating both stiffness and stretch. Using the model, we evaluate the role of mechanical signaling in homeostasis and disease progression during self-healing and fibrosis, respectively. We develop the model such that there is a fibrotic threshold near which the network tends towards instability and fibrosis or below which the network tends to heal. The healing response is due to the stretch signal, whereas the fibrotic response occurs when the stiffness signal overpowers the stretch signal, creating a positive feedback loop. We also find that by changing the proportional weights of the stretch and stiffness signals, we observe heterogeneity in pathological network structure similar to that seen in human IPF tissue. The system also shows emergent behavior and bifurcations: whether the network will heal or turn fibrotic depends on the initial network organization of the damage, clearly demonstrating structure’s pivotal role in healing or fibrosis of the overall network. In summary, these results strongly suggest that the mechanical signaling present in the lungs combined with network effects contribute to both homeostasis and disease progression.

## Introduction

Fibroblasts and myofibroblasts play an essential role in tissue healing and are disrupted in pulmonary fibrosis (PF), a deadly disease. While healing represents the process whereby tissue returns to its homeostatic condition, PF involves the aberrant remodeling of parenchymal tissue, which produces diverse characteristic structures such as honeycombing and dense fibrotic patches (Tanabe et al., 2020). The rate at which PF progresses is also diverse. For example, idiopathic pulmonary fibrosis (IPF) results in an average lifespan of 3–5 years after diagnosis and has no treatment (Nathan et al., 2011). In contrast, severe post-COVID pulmonary fibrosis (PCPF) sometimes resolves (Lorx et al., 2022), and PF caused by ventilation-induced injuries during acute respiratory distress syndrome can also heal either partially or completely (Herridge et al., 2011). Nevertheless, the mechanisms leading to these different forms of PF, and exactly how fibroblasts and myofibroblasts are involved, are still not fully understood.

It is well established that substrate stiffness can affect how fibroblasts deposit collagen and proliferate, which can result in positive feedback leading to fibrosis (Liu et al., 2010; Moore and Herzog, 2013; Lin et al., 2017; Tschumperlin et al., 2018). In a mechanically dynamic organ such as the lung, the mechanical stimulus provided by cyclical stretch also affects fibroblast behavior by, for example, activating stretch-regulated channels in the cell membrane (Murata et al., 2014). Stretch also contributes to the release of latent TGF-β (Klingberg, F. et al., 2014; Ezzo and Hinz, 2023; Hinz, 2009), a cytokine essential for wound healing that is involved in the transformation from fibroblasts to myofibroblasts (Hinz, 2009). Signaling pathways such as YAP and MRTF-A have also been linked to fibroblast proliferation in response to cyclical stretch (Cui et al., 2015). YAP is essential in regulating ECM deposition (Liu et al., 2015), while MRTF-A contributes to the transition of fibroblasts into a myofibroblast phenotype (Wagner et al., 2020). These various mechanotransduction mechanisms are not only important regulators of fibroblast differentiation and behavior, but also contribute to disease progression in PF.

The above considerations suggest that lung parenchymal homeostasis is maintained via a balance of mechanotransduction signals related to substrate stiffness and stretch, and that when the system is perturbed sufficiently, the self-healing nature of tissue is overpowered by progressive stiffening, leading to PF. We have previously developed several computational models of PF pathogenesis that support this hypothesis (Wellman et al., 2018; Suki et al., 2020; Suki et al., 2020; Hall et al., 2023). In these models, elastic spring networks mimic the structure and mechanics of the parenchymal tissue, while autonomous agents mimic the cellular processes that act on the tissue. These previous models, however, have not investigated how mechanical stretch affects fibroblast and myofibroblast behavior and the emergent pathology that results, and therefore they fail to capture critical features of a dynamic organ like the lung. Therefore, in the present study, we developed an agent-based model (ABM) operating on a non-uniform network of elastic springs to investigate how stretch and substrate stiffness interact to influence fibroblast and myofibroblast behavior, and how this potentially leads to the development of PF.

## Materials and methods

We developed a computational model in which agents, which represent fibroblasts, move around randomly on a spring network that represents the extracellular matrix (ECM) of the lung. Agents become activated according to the strains and stiffnesses of the springs they traverse during their travels. Positive activation causes an agent to increase the stiffness of any spring it crosses (representing deposition of collagen). A positively activated agent also has a higher likelihood of proliferating. Negative activation causes an agent to reduce the stiffness of any spring it crosses (representing digestion of collagen), and to have a higher likelihood of death. An activation of zero corresponds to an agent at equilibrium.

First, a set of discrete difference equations were developed to drive the behavior of the agents, and the behavior of these equations was evaluated independent of a network. Then, these equations were applied to agents on a network to observe what emergent properties the network would provide.

### Agent-spring interactions

The springs in our network model of ECM are Hookean, each with its own spring constant K . If we assume that the value of K for a given spring is proportional to its cross-sectional area, A , as would be the case for a strip of lung tissue, then

$$K=\frac{EA}{L_{0}}$$ (1)

where *E* is Young’s modulus and *L*_*0*_ is the resting length of the spring.

Whenever an agent traverses a spring during a time step, it evaluates K for that spring (corresponding to the way that cells probe substrate stiffness via their integrins). K then makes a contribution to the agent’s level of activation, $a_{K}$ , for the subsequent time step according to

aK=KβKβ+1=EALoEAtLoβEALoEAtLoβ+1 (2)

where $A_{t}$ is a cross-sectional area constant near which the system becomes unstable. We assume *E* and *L*_*0*_ are both constant so that variations in spring stiffness are determined solely by variations in cross-sectional area. The uniform *L*_*0*_ arises from the assumption that the cells themselves all have similar length, and regardless of the length of the fibers they are interacting with, the length that the cells, and hence the corresponding agents, perceive is limited to their own length, *L*_*0*_, which is constant. With these assumptions, Eq. 2 simplifies to

$$a_{K}=\frac{{\left(\frac{A}{A_{t}}\right)}^{\beta }}{{\left(\frac{A}{A_{t}}\right)}^{\beta }+1}$$ (3)

Eq. 3 is a Hill function (Hill, 1910), which makes $a_{K}$ self-limited. Thus, although $a_{K}$ is always positive, it cannot increase without limit, as must be the case since fibroblasts have finite resources.

Similarly, the strain, ε , of the spring that an agent traverses also makes a contribution to the level of agent activation, representing activation via the release of ECM-bound latent TGF-beta, (Hinz, 2009; Ezzo and Hinz, 2023), stretch-sensitive channels (Murata et al., 2014), and signaling pathways such as YAP and MRTF-A (Cui et al., 2015). This contribution is proportional to

$$a_{\varepsilon }=\frac{{\left(\frac{\varepsilon }{\varepsilon_{s}}\right)}^{\gamma }}{{\left(\frac{\varepsilon }{\varepsilon_{s}}\right)}^{\gamma }+1}$$ (4)

where $\varepsilon_{s}$ is the target (homeostatic) strain at steady-state and we arbitrarily set γ=1 .

The total activation of an agent when it traverses a spring of stiffness K and strain ε is a weighted sum of the contributions from stiffness and strain thus:

$$a=w_{1}a_{\varepsilon }+w_{2}a_{K}-\;c$$ (5)

where *c* is a constant that causes *a* = 0 when *ε* = *ε_s_* and *A* = *A*_0_, and the weighting factors $w_{1}$ and $w_{2}$ determine the relative influences of strain and stiffness, respectively, on agent activation, and can be considered as reflecting genetic and/or environmental predispositions towards different forms of fibrosis. For all the simulations in the present study, we set $w_{1}=1$ and varied $w_{2}$ . The total activation can thus be either positive or negative described by a Hill function that is self-limited.

At homeostasis, $\varepsilon =\varepsilon_{s}$ , $A=A_{0}$ , and a=0 . Substituting Eqs 3, 4 into Eq. 5 under these conditions gives the constant c as thus:

$$c=w_{1}\frac{{\left(\frac{\varepsilon_{s}}{\varepsilon_{s}}\right)}^{\gamma }}{{\left(\frac{\varepsilon_{s}}{\varepsilon_{s}}\right)}^{\gamma }+1}+w_{2}\frac{{\left(\frac{A_{0}}{A_{t}}\right)}^{\beta }}{{\left(\frac{A_{0}}{A_{t}}\right)}^{\beta }+1}=\frac{1}{2}w_{1}+w_{2}\frac{{\left(\frac{A_{0}}{A_{t}}\right)}^{\beta }}{{\left(\frac{A_{0}}{A_{t}}\right)}^{\beta }+1}$$ (6)

The tissue at homeostasis is well below the fibrotic threshold, meaning $A_{0}\ll A_{t}$ , in which case the second term in Eq. 5 is much less than the first term (for values of $w_{2}$ that are of a similar order of magnitude to $w_{1}$ ). This gives c≈0.5 . We found by trial and error during initial testing that the stiffness Hill function (Eq. 3) needs to be steep at low values of A in order to obtain both the healing and fibrotic responses, so we set β=3 .

At each time step, j , each agent modifies the value of A for the spring it traverses, so A is updated according to

$$A_{j+1}=A_{j}+PD_{j}a_{j}$$ (7)

where *P* is a scaling constant, and $D_{j}$ is the current density of agents on the network, defined as the average number of agents per spring. Note that in the difference equations, $D_{j}$ is modeled explicitly (see Eq. 10 below), while in the network model, $D_{j}$ changes through the probabilistic appearance and disappearance of discrete agents. Each spring in the network model has an individual $A_{j}$ , and each agent has an individual $a_{j}$ that is modulated by the spring it crosses, so $D_{j}$ can be considered as the probability that an agent will cross a spring in the network during a given iteration. The values of a for springs not traversed by an agent during the j ^th^ time step do not change.

Since a may be either positive or negative, $∆A_{j}=PD_{j}a_{j}$ may also be positive (corresponding to collagen deposition) or negative (corresponding to tissue digestion). If repeated application of Eq. 7 ever produces the condition $A_{j}\leq 0$ , the spring in question is considered to have ruptured and is removed from the network so that it no longer provides a migration pathway for agents. Isolated springs around which all connecting springs have ruptured are also removed. Similarly, agents are removed if they become stranded on nodes that are unconnected to any springs.

For the set of difference equations, it is necessary to model the strain of a hypothetical spring. Therefore, a spring under a constant external force was utilized, and the strain at any given step for a change in $A_{j}$ was modeled thus:

$$\varepsilon_{j+1}=\varepsilon_{j}+\frac{A_{0}\varepsilon_{s}}{A_{j}}$$ (8)

The corresponding agent activation at the j ^th^ time step is, from Eq. 5, updated according to

$$a_{j+1}=a_{j}+r\left(w_{1}a_{\varepsilon ,j}+w_{2}a_{K,j}-a_{j}-\;c\right)=a_{j}\left(1-r\right)+r\left(w_{1}a_{\varepsilon ,j}+w_{2}a_{K,j}-\;c\right)$$ (9)

where $a_{K,j}$ and $a_{\varepsilon ,j}$ are given by Eqs 3, 4, respectively, for each agent according to the stiffness and strain of the spring they just crossed. The constant 0≤r≤1 defines the memory of the agent (Wellman et al., 2018; Suki et al., 2020). Having r=1 means that activation is updated to a new value that depends only on the current values of spring stiffness and strain. When r<1 , activation remembers a fraction of its previous value. For this model, we used r=0.5 . (For further details, see Supplementary S.1–S.5).

Although we do not define agents as corresponding explicitly to either fibroblasts or myofibroblasts, agents with a close to zero are considered to be fibroblasts, while agents with a approaching the maximum possible value are considered to be myofibroblasts. Agents with intermediate values of a are considered to be in transition. Both fibroblasts and myofibroblasts increase in number in PF (Moore and Herzog, 2013), although the source of myofibroblasts is still debated (Moore and Herzog, 2013). For simplicity, we assumed that myofibroblasts arise only via differentiation of fibroblasts, while fibroblasts can migrate into the lung tissue from surrounding regions. Accordingly, agent density is updated according to (see Supplement for further details):

$$D_{j+1}=D_{j}+\frac{p_{1}n}{s}+\left[p_{3}a_{j}\;-\;p_{2}\right]D_{j}$$ (10)

where $p_{1}$ is the probability that an agent will appear spontaneously at any given node in a given time step (representing migration from outside the tissue), *n* is the number of nodes in the network, *s* is the number of springs in the network, $p_{2}$ is the probability that any given agent will die at a given time step, and $p_{3}$ is the probability that an agent will divide into two agents. (For further details, see Supplementary S.6–S.11). This equation has two steady-states for which the change in $D_{j}$ is zero, one for the fibrotic response and one for the self-healing response. When $a_{j}=0$ , the change in $D_{j}$ approaches zero exponentially for the homeostatic state. If $a_{j}$ is at its maximum value, the change in $D_{j}$ will also approach zero for the fully fibrotic state, representing how activated myofibroblasts resist apoptosis (Ezzo and Hinz, 2023). The upper limit on $D_{j}$ represents the finite carrying capacity of tissue for fibroproliferative cells.

Eqs 7–10 comprise a set of coupled finite difference equations that represent the interactions between a collection of agents and the springs they traverse.

### Spring network representation of ECM

At each time step as the model is run, these equations are applied to each agent and to each spring that is traversed by an agent. Of course, not all springs in the network are necessarily visited by an agent in a given time step, but all springs will be modified if the model is run for long enough. The overall behavior of the model, therefore, is determined not only by the dynamics inherent in Eqs 7–10 but also by the local dynamics of agent movement over the network.

We created a non-uniform spring network to represent the parenchymal tissue using a method we have described previously (Hall et al., 2023). Briefly, Poisson Disk Sampling was used to create a semi-organized 2D Voronoi diagram. The edges of the diagram represented springs with uniform values of A and E , with *L*_*0*_ proportional to edge length, allowing K for each spring to be calculated from Eq. 1. This produced a network with uniform strain. This initial configuration of the network was not at equilibrium, however. The equilibrium configuration was found by simulated annealing in which the nodes were moved in random directions by progressively decreasing amounts until the net force on each node was minimized, similar to the energy optimization method we have described previously (Cavalcante et al., 2005; Hermann, 2021; Hall et al., 2023) As the model simulations progressed, the continually changing values of A throughout the network caused its equilibrium configuration to change. Simulated annealing was used to determine the new configuration at each time step.

In the network, the strain on each spring is a result of the solved equilibrium state of the network, and is solved at each iteration. This static strain is used as an approximation of the cyclical strain to limit computational requirements. This equilibrium strain is different from the single element approximation from Eq. 8 due to heterogeneity in strain caused by network effects. In order to recapitulate the reduction in lung volume that occurs in PF as the lungs get stiffer (Plantier et al., 2018), we applied non-fixed boundary conditions to the network by applying external forces to the boundary of the network that were equal to the reaction forces of the pre-stressed network. These reaction forces were kept constant throughout the simulations. Tissue injury was simulated by modifying the value of A for a given percent of springs in the network.

Network functionality was quantified in terms of a strain-energy function previously described in Kim et al., 2023. Briefly, the network underwent 5 accumulating stretches of 2% strain each, and the energies in each spring in the network following each stretch were summed. The resulting strain-energy plot was fit with a second-order polynomial function, and the second order constant was as a measure of overall network stiffness. Each network was normalized by its initial stiffness such that the changes in stiffness across all networks were comparable.

We created synthetic histologic images of lung tissue by drawing the networks with edge thicknesses proportional to A . The synthetic images were compared to histologic images of lung tissue with IPF obtained from Tanabe et al., 2020.

At least nine network were run for each $w_{2}$ value. Each model simulation was run for 2000 iterations, which corresponds to a duration in real time of approximately 5 months (Supplementary Eq. S12) based on measured migration speeds of fibroblasts on two-dimensional gels (Tschumperlin, 2013). Table 1 lists all the model parameters, including those assigned values following initial model testing based on the recapitulation of reasonable physiologic behavior. Those not assigned specific values as discussed above were assigned values following initial model testing based on the recapitulation of reasonable physiologic behavior.

**TABLE 1:** List of model parameters and variables. Values are given to symbols representing parameters.

Parameter	Meaning	Value
***a***	Activation	∼
***A***	Cross-sectional Area	∼
***A***_***o***_	Initial cross-sectional area	1
***A***_***T***_	Cross-sectional area fibrotic threshold	10
***D***	Agent Density (agents/node)	∼
***D***_***o***_	Initial Agent Density	0.3
***D***_***max***_	Maximum Agent Density	3
**ε**	Spring Strain	∼
**ε**_**o**_	Initial Spring Strain	1
**ε**_**s**_	Target Spring Strain	1
$w_{1}$	Strain weight	1
$w_{2}$	Stiffness weight	1–3
**r**	Agent Memory	0.5
**γ**	Steepness of Strain Hill Function	1
**β**	Steepness of Stiffness Hill Function	3
**c**	Steady-State Constant	∼
**P**	Production Constant	0.2
$p_{1}$	Probability of new Agent appearing	0.01
$p_{2}$	Probability of Agent Dying	∼
$p_{3}$	Probability of Agents Dividing	∼

## Results

**FIGURE 1:**
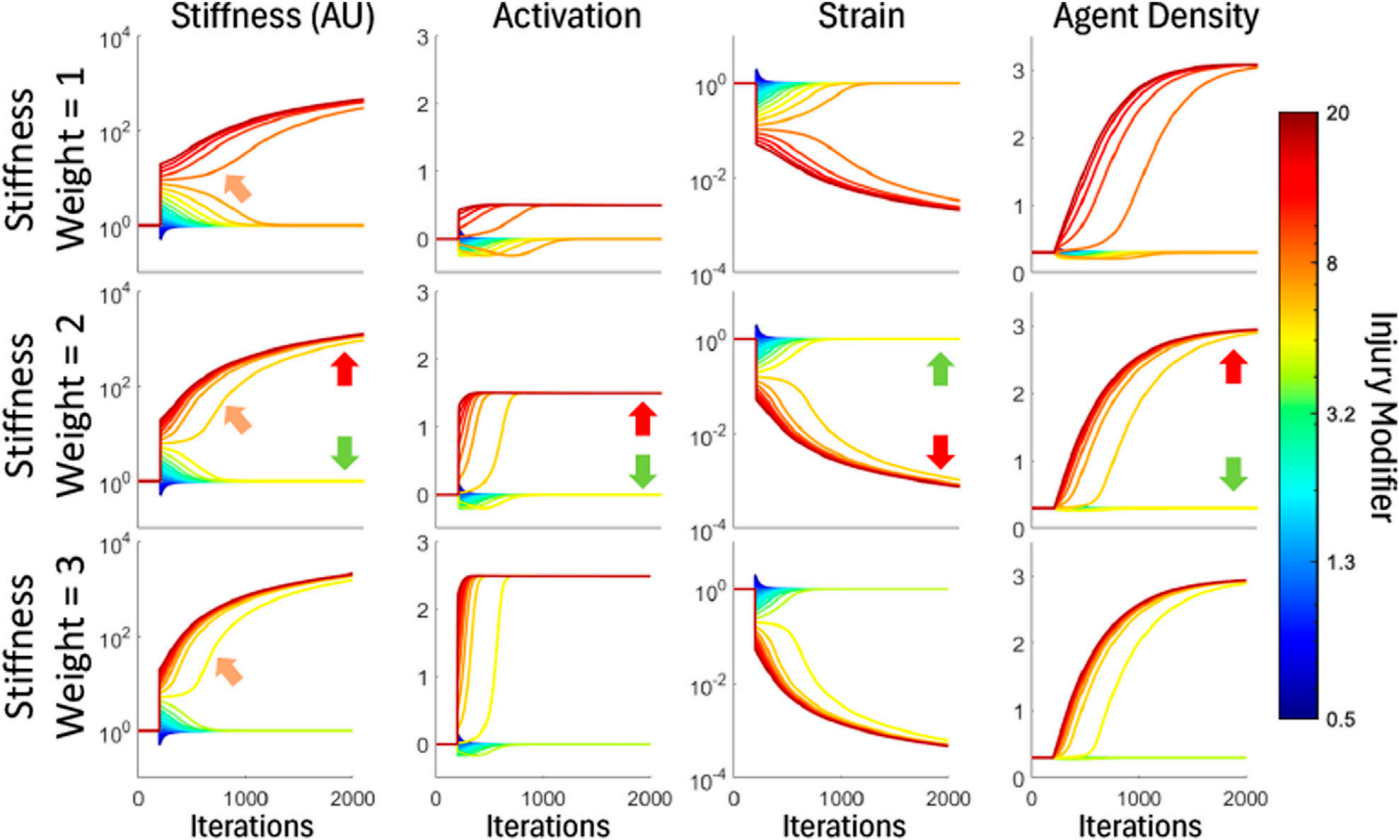
Difference equations responses to perturbations. Each color represents a response to a pertrubation modifying the cross-sectional area (*A*), ranging from softening (blue) to stiffening (red). Each row corresponds to increasingly fibrotic reactions due to increasing stiffness weight ( $w_{2}$ ). The first column shows the cross-sectional area responses to perturbations, the second column shows the activation (*a*) response, the third column shows strain (ε) response, and the fourth column shows the agent density (*D*) response. In each response, we see healing, where the parameter returns to its initial value, or fibrosis where the parameter either saturates to a non-initial value, or changes without bound. Examples of healing and fibrosis in the Moderate Sensitivity are indicated by green arrows and red arrows respectively. These responses show the conditional stability of this self-healing model. The orange arrows in the first column show the lowest injury modifier that results in fibrosis, and as the sensitivity increases, the lowest injury modifier decreases, showing a lower threshold for a fibrotic response systems with a greater stiffness weight.

In order to understand the dynamics inherent to the difference equations (Eqs 7–10), the initial conditions provided in Table 1 were used, and the system was run for 2000 iterations. Injury was inflicted by altering the value of A to make the spring either softer or stiffer. We found that this simple system is capable of exhibiting both self-healing and fibrotic responses, as shown in Figure 1. When the system is in the process of healing, the key variables K , a , ε , and D all return asymptotically to their steady-state (homeostatic) values at rates that depend on the severity of injury. This applies when injury involves either stiffening or softening of the spring. However, there is a threshold level of fibrotic injury above which the system does not heal. Instead, a approaches an elevated maximum value that depends on the values of $w_{1}$ and $w_{2}$ in Eq. 7. In addition, A grows without limit, ε drops toward zero, and *D* approaches its maximum allowable value. We found this bifurcation behavior to be qualitatively similar for three different weight values of $w_{2}$ , demonstrating a degree of model robustness in terms of the relative influences of genetic and environmental factors on fibrosis development (Eq. 8). However, we found the system becomes unstable when $w_{2}=0.5$ because this causes D to grow infinitely due to dividing by zero in the $p_{3}$ parameter (Supplementary Eq. S11). On the other hand, $w_{2}<0.5$ , the system always heals because the fibrotic response is always weaker than the healing response. Thus, for the model to have the capacity to exhibit both fibrotic and self-healing responses to injury, we must have the condition that $w_{2}>0.5$ ( $w_{1}$ being maintained at a value of 1).

**FIGURE 2:**
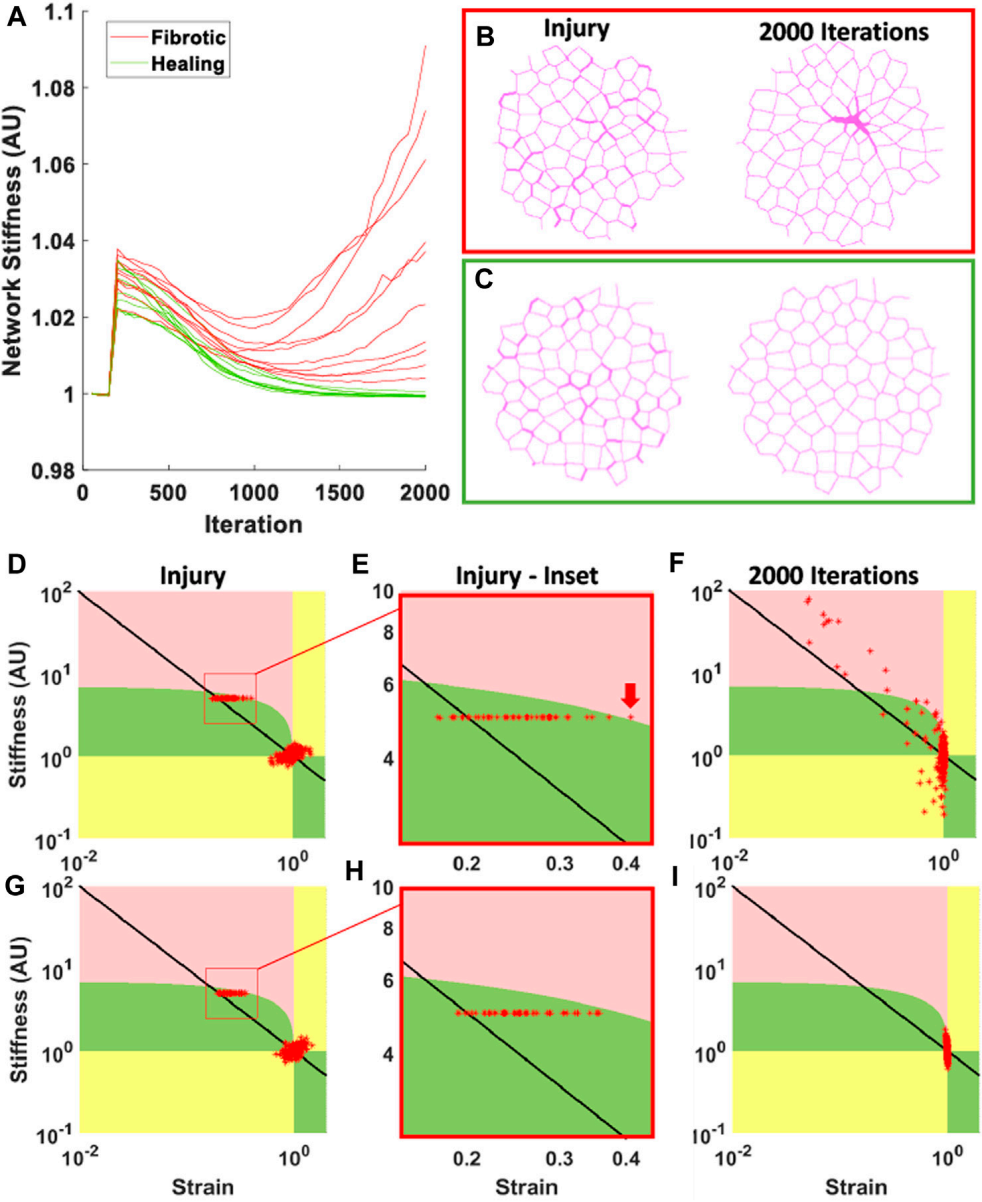
Diverging behavior resulting from network effects. **(A)** Result of 20 networks where 20% of springs were stiffened to a cross-sectional area (*A*) 5x the initial cross-sectional area. Green plots indicate self-healing, returning near to the original stiffness, red plots indicate fibrotic responses. **(B)** and **(C)** Examples of networks with fibrotic and healing responses respectively. Left shows networks at injury, and right shows networks at 2000 iterations. **(D–I)** show network phase diagrams corresponding to the networks shown in **(B)** and **(C)**, where green is self-healing phase, pink is fibrotic phase, yellow is mixed-behavior phase, and steady-state is at the point (1,1). Each red star represents the state of a given spring, and the black line represents the behavior of the difference equations. **(D)** and **(G)** show spring states at injury, where injured springs are shown in red boxes. **(E)** and **(H)** show these red boxes indicated in panels **(D)** and **(G)**, and the red arrow indicates a spring that has crossed into the fibrotic phase, causing that network to become fibrotic at 2000 iterations. **(F)** and **(I)** show spring state progressions in panels **(D)** and **(G)** at 2000 iterations. **(D)** has turned fibrotic, as many springs can be seen in the fibrotic phase and away from steady-state, while **(I)** has healed, as all the springs have returned to near the steady-state. For additional details related to panel D, see Supplementary Figure S4.

Next, we evaluated the behavior of the complete spring network model with the difference equations applied to mobile agents. As with the difference equations, we found that the complete model is also able to self-heal or become fibrotic depending on the severity of the tissue injury, with dynamics that depend on both the amount and severity of injury (Figures 2A–C). The Network heals following all softening injuries and those minor stiffening injuries that are well below the fibrotic threshold, while the network becomes progressively fibrotic for major stiffening injuries clearly above the fibrotic threshold.

While the difference equations exhibit a sharp bifurcation behavior between healing and fibrosis, the network model has a range of moderate injury that can lead to either healing of fibrosis. A fibrotic response in this range can be caused, for example, by some springs that are initially very close to the fibrotic threshold being pushed over the threshold by the evolving behavior of nearby springs, as illustrated in Figures 2B–F.

**FIGURE 3:**
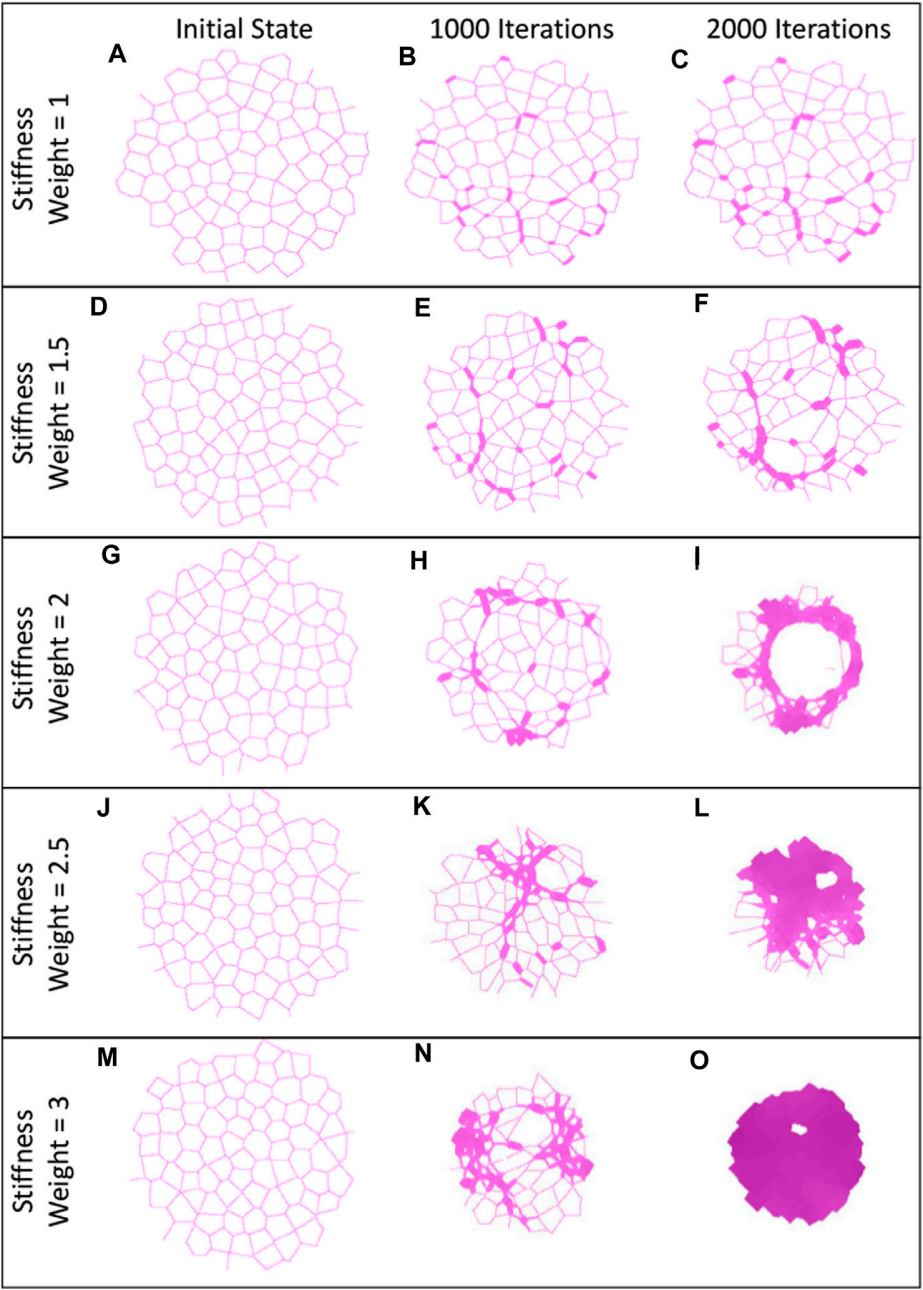
Examples of heterogeneous fibrotic structures. Initially, each of these networks had 10% of the springs stiffened to 10x the initial cross-sectional area. Each row corresponds to increasingly fibrotic reactions due to increasing stiffness weight ( $w_{2}$ ). The first column **(A, D, G, J and M)** shows networks at their initial healthy state. The second column **(B, E, H, K and O)** shows networks at 1000 iterations. The third column **(C, F, I, L and P)** shows networks at 2000 iterations. The third column exhibits many fibrotic structures, such as sparse fibrotic foci in **(C)**, honeycombing in **(I)**, and a dense fibrotic patch in **(P)**.

**FIGURE 4:**
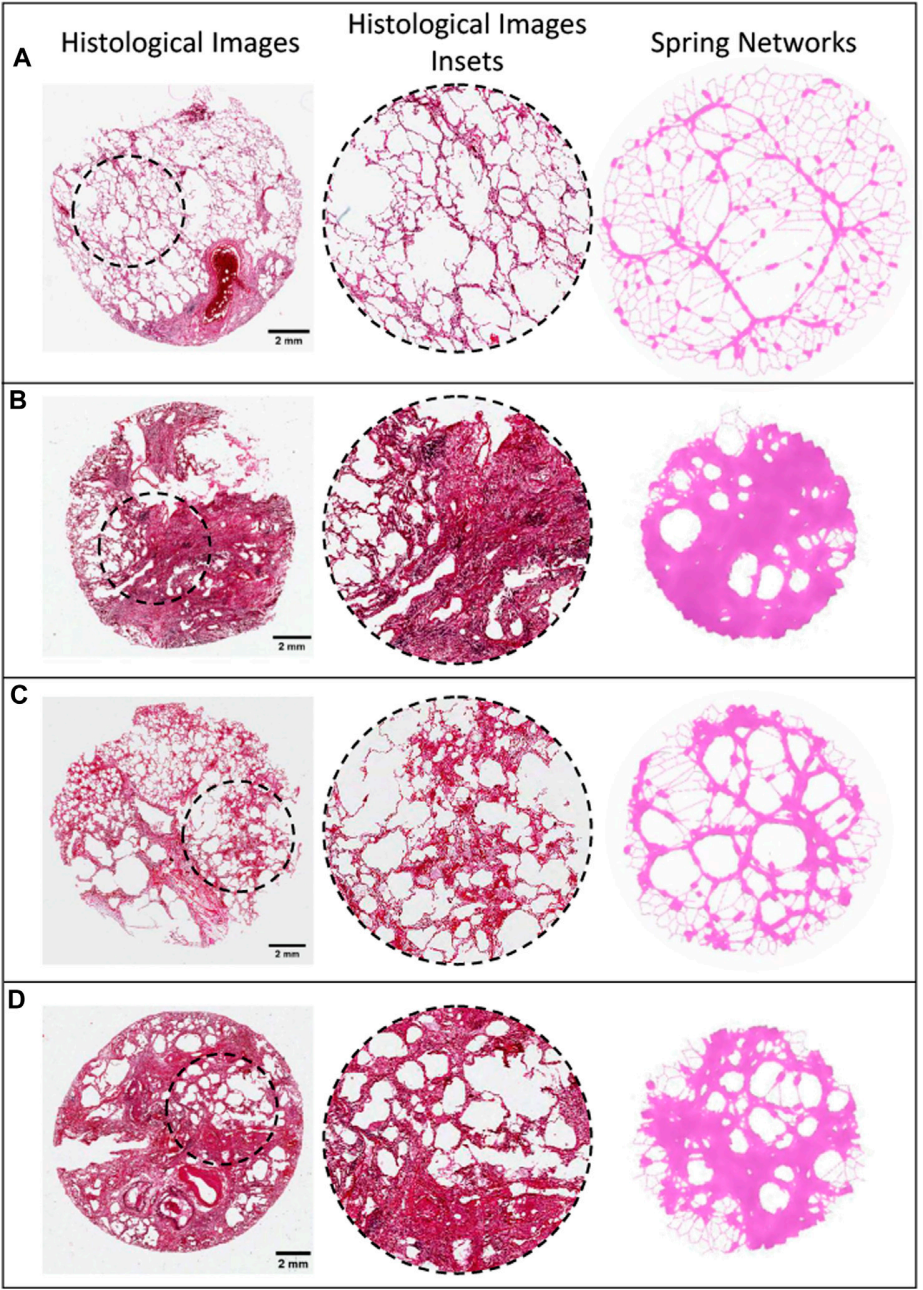
Comparison of IPF histological images and spring networks. The first column shows histological slices of IPF tissue with differing structures, and the second column shows enlarged sections from these slices (Modified with Permission from Tanabe et al., 2020). The third column shows spring networks of varying sensitivity at varying stages of progression that are similar in structure to the images on the same row **(A–C)**.

We found that $w_{2}$ has a marked effect on fibrotic phenotype as characterized by fibrotic sparseness, honeycombing, and growth of dense fibrotic patches. Figure 3 illustrates how these features progress as $w_{2}$ increases relative to $w_{1}$ . Figure 4 shows that these features are highly reminiscent of histologic features seen in actual histologic images of lung parenchyma at varying stages of disease progression.

**FIGURE 5:**
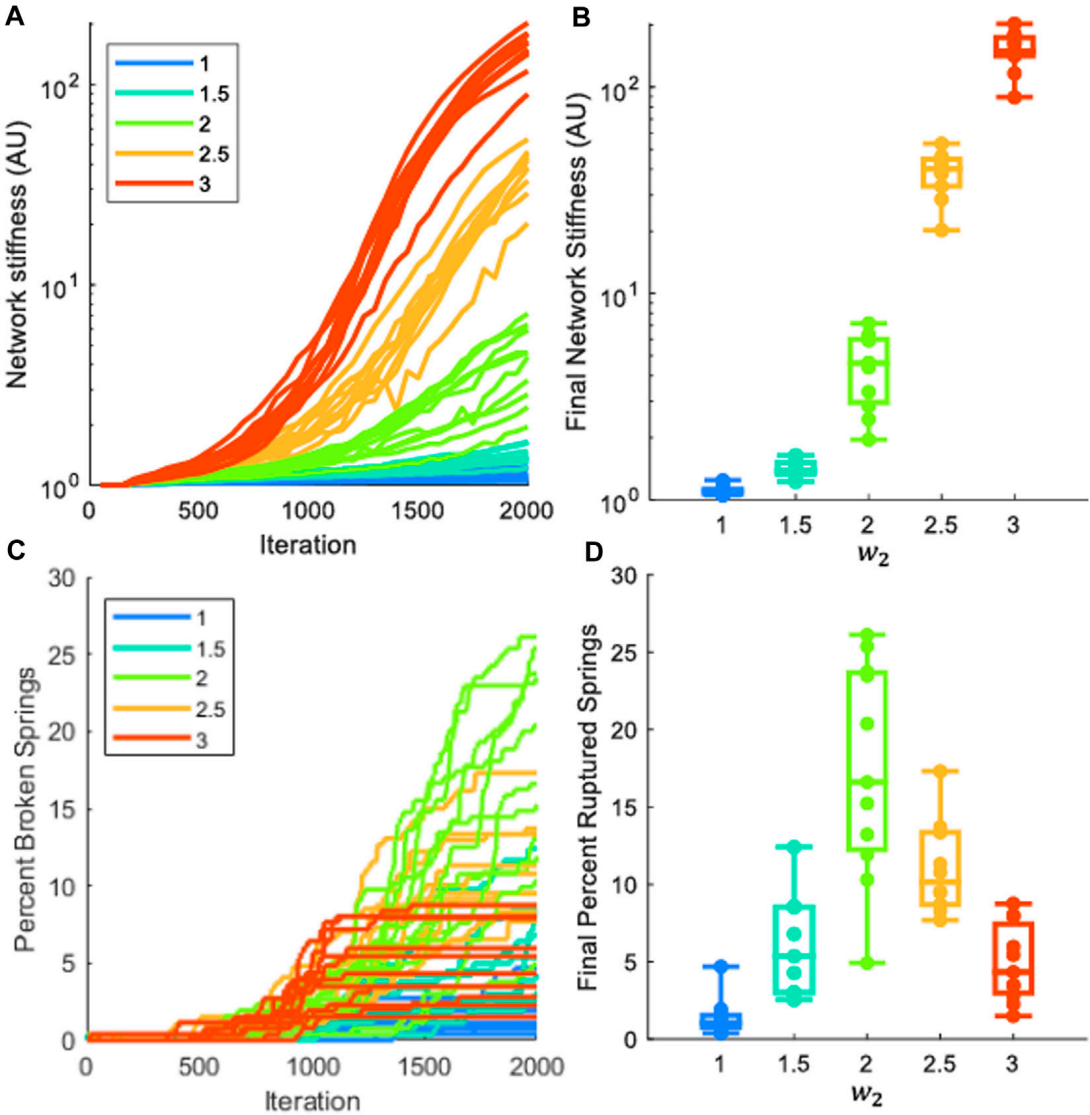
Analysis of mechanics in fibrotic network progression. Each network had 10% of the springs stiffened by 10x the initial cross-sectional area. Each color represents a different stiffness weight ( $w_{2}$ ), and each sensitivity was tested with at least nine networks. **(A)** Network stiffness as progression occurs **(B)** Box plot of final stiffness distributions. Populations not of same distribution, p << 0.001. **(C)** Percent ruptured springs as progression occurs **(D)** Box plot of final ruptured springs distributions. Populations not of same distribution, p << 0.001.

Figure 5 shows that the progression of fibrosis in the model is characterized by progressive increases in network stiffness (Figure 5A). The networks that were weighted toward a greater fibrotic response (greater values of $w_{2}$ in Eq. 8) developed fibrosis more quickly. A Kruskal-Wallis test determined that the populations of responses in network stiffness obtained under the different conditions shown were not of the same distribution (Figure 5B, p << 0.001). We also determined the percentage of springs rupturing, relative to the initial number of springs, as fibrosis progressed (Figure 5C), and found that the final populations were not all of the same distribution (Kruskal-Wallis test, p << 0.001, Figure 5D). Interestingly, the percentage of broken springs does not exhibit a monotonic relationship with $w_{2}$ (Figures 5C,D); there is little breakage when the propensity for fibrosis is low ( $w_{2}$ is small), but breakage also decreases as $w_{2}$ increases above a certain level.

**FIGURE 6:**
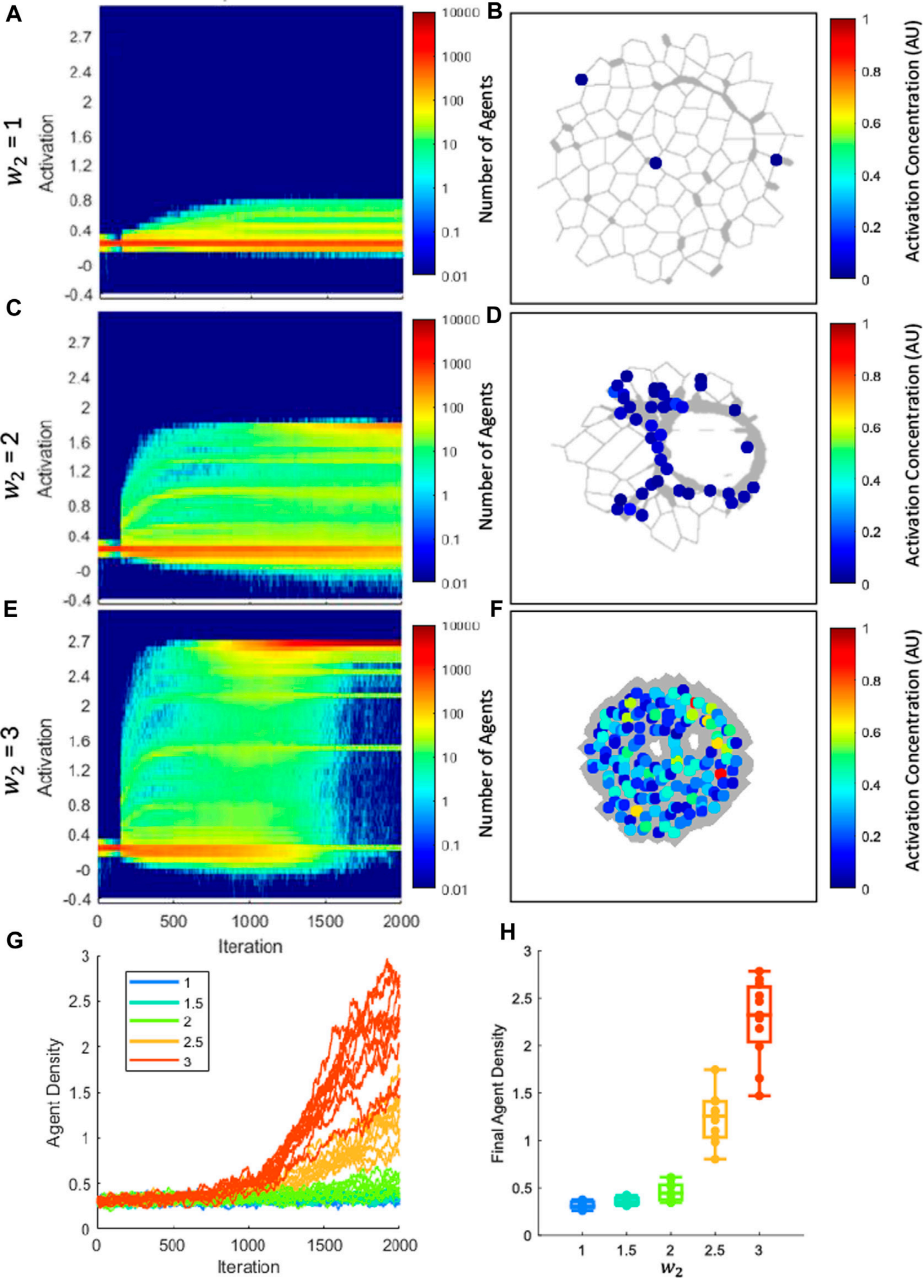
Differentiation of agents from early fibroblast to myofibroblast phenotype. **(A, C, E)** show activation distribution and number of agents as disease progresses, where color indicates number of agents at a given activation. Each panel is the sum of all agents used in all networks for the listed sensitivity. In **(A)**, the majority of the population stays at the initial activation, indicating that most agents stay in a fibroblast phenotype. In **(C)** a second population of higher-activated agents is present at 2000 iterations that indicates partial differentiation into myofibroblasts. In **(E)** the low-activation fibroblast population nearly disappears by 2000 iterations, while the high-activation myofibroblast population has become dominant, indicating differentiation. **(B, D, F)** Local activation plots superimposed on networks. As fibrosis progresses, it is clear that agents cluster in fibrotic regions and are highly activated. **(G)** The density vs. iteration for all network simulations. Each color represents a different stiffness weight ( $w_{2}$ ). **(H)** Box plots of final agent densities. Populations not of same distribution, p << 0.001.

Figure 6 shows how agent activation level and density vary across the network as a function of time. The parameters of all agents across every simulation of matching $w_{2}$ were pooled for analysis in Figures 6A,C, E. In the networks with the smallest values of $w_{2}$ , agents remained in low activation (Figure 6A). There were too few highly-activated agents to generate any particular assembly pattern across the tissue (Figure 6B). For intermediate values of $w_{2}$ , a more substantial secondary population of highly activated agents appeared (Figure 6C), and these were concentrated near fibrotic regions of the network (Figure 6D). When the value of $w_{2}$ was high, the population of low-activation agents essentially disappeared, while the activated population increased both in number and activation level (Figure 6E) and were so numerous that again no particular clustering pattern was evident (Figure 6F). The residual low-activation agents are largely a result of the agents appearing spontaneously at nodes. In an analysis of the final population of agents across all models, a Kruskal-Wallis test determined the populations were not of the same distribution with p << 0.001 (Figure 6H).

## Discussion

Pulmonary fibrosis is a deadly disease characterized by micromechanical tissue changes. The prevailing hypothesis identifies fibroblasts and myofibroblasts as the key mechanosensory cells involved in disease progression. These cells respond to tissue stiffening by activation, leading to extracellular matrix (ECM) deposition. Less well known is the cellular responses to stretch. Accordingly, we developed an ABM-spring network hybrid model that includes both stiffness and stretch motivated behaviors, and evaluated the resulting structural and mechanical properties of the resulting networks. The main results can be summarized as follows: 1) strain-driven mechanotransduction is important in the inherent self-healing ability of the tissue; 2) sufficient regional stiffening prohibits self-healing and the tissue becomes fibrotic via a positive feedback loop; 3) there is a bifurcation in the system because, depending on the initial configuration of regional stiffening, the system can self-heal or turn fibrotic; and 4) fibrotic phenotype depends on the balance between stiffness-dependent and stretch-related mechanosensitive pathways.

### Comparison to previous models

Within the processes of self-healing and fibrosis are myriad interactions between cell types and systems that result in the behaviors and structures observable in tissue (Ezzo and Hinz, 2003; Moore and Herzog, 2013; Pally and Naba, 2024). Many of these interactions are not fully understood, therefore in this paper we did not endeavor to include each of these, but instead created a lumped-parameter model (Brown et al., 2011) that focuses on broad behaviors such as the deposition and digestion of tissue in response to stiffness and stretch. We also focused primarily on 2 cell types, specifically the transition of fibroblasts into myofibroblasts. This lumped-parameter model serves as a means of broadly understanding the contribution of stretch-mediated behavior in addition to the previously established stiffness-mediated behaviors in fibroblast-like agents (Wellman et al., 2018).

In a previous PF model, Wellman et al., 2018 developed a stiffness-motivated migration ABM on a 2D hexagonal spring network with agents’ behavior defined by spring stiffness. The Wellman model included wall rupture, and found that the distribution of Low Attenuation Areas (LAAs) matched that in CT scans, however, it was not able to heal itself or maintain steady-state; that is to say, the Wellman model could only progress towards fibrosis. Suki et al., 2020 developed a self-healing model on a 2D hexagonal network, however induced fibrosis required a constant perturbation of the agents, rather than fibrosis induced from a single insult. Bates et al., 2007 and Hall et al., 2023 explored fibrosis on 2D hexagonal networks and 3D non-uniform networks, respectively, and both observed clustering and mechanics seen in PF, but neither included rupture, gradient in agent behavior, or self-healing.

While many of these previous studies have utilized uniform networks, such as square and hexagonal lattices, it has been shown that non-uniform networks have more geometric and mechanical similarity to lung tissue (Hall et al., 2023), therefore a non-uniform network was used for this model. Comparison of broad characteristics of this fibrotic model appeared similar between non-uniform and hexagonal networks (Supplementary Figure S3). Furthermore, previous models have modeled agents as preferentially migrating towards stiffer substrates based on fibroblast behavior (Liu et al., 2010; Wellman et al., 2018), however, there is the potential for cells to migrate towards high-strain or injured tissue (López-Martínez et al., 2018). To maintain the simplicity of the model, random migration was used in all cases.

Each of these previous models contributed key insights into the mechanics of lung tissue and PF pathogenesis, however, each had key limitations. Furthermore, no previous computational models have investigated how mechanical stretch affects fibroblast and myofibroblast behavior, and the resulting emergent structural behaviors. This indicates that a model that only includes behavior defined by stiffness does not capture all the mechanical properties inherent to a dynamic organ like the lung. The model developed in this work was able to recapitulate many of the essential behaviors, such as both healing and fibrosis, and structures such as honeycombing and fibrotic clustering, while introducing new behaviors, such as shrinkage and clear differentiation. These behaviors are explored more thoroughly in the following sections.

### Stretch optimization and structural heterogeneity

A fibroblast’s primary function is the maintenance of the extracellular matrix (D’Urso and Kurniawan, 2020) through the strategic deposition and digestion of tissue. A key mechanism by which fibroblasts communicate with, migrate through, deform, and are deformed by their environment is through focal adhesions (Mierke et al., 2017; Stutchbury et al., 2017; Tschumperlin et al., 2018). It is known that fibroblasts are able to react in response to a material’s stiffness by depositing more material, and it is hypothesized that the cell is able to measure local tissue stiffness by active contraction (Engler et al., 2006). Together, this led to our first rule: the agent activation has a positive relationship with local stiffness. We also assume that fibroblast cannot perceive anything beyond the grasp of its focal adhesions. In the context of a spring, this means that the agent cannot perceive the length of the whole spring, only the length of the cell. Therefore, for a measured spring constant, the perceived *L*_*0*_ would be approximately the length of the fibroblast itself, and can be considered as a constant for all agents. Furthermore, for simplicity, we make an assumption that the composition of the tissue is mostly collagen, and that therefore the Young’s modulus *E* of the tissue is uniform. This is what leads to the simplification from Eqs 3, 4.

Next, we must also consider the fibroblast’s ability to perceive stretch. Fibroblasts are affected by stretch via the release of ECM-bound latent TGF-beta, which supports the transition from fibroblasts to myofibroblasts (Hinz, 2009; Ezzo and Hinz, 2023), activates stretch-sensitive channels (Murata et al., 2014), and affects signaling pathways such as YAP and MRTF-A (Cui et al., 2015). Therefore, higher stretch may increase cell activation and lead to increase in collagen deposition (Tschumperlin et al., 2018). In the lungs, the periodic stretching provides a constant signal to the fibroblasts, and we hypothesize that there is an optimal range of stretch that the fibroblasts expect, such that when the stretch is too high, collagen is deposited to stiffen the material and decrease stretch, and when stretch is too low, the tissue is digested to increase stretch, creating a negative-feedback healing response. This led to our second rule: agents increase activation in response to local stretch. This contrasts with the notion that fibroblasts maintain the stiffness of a material. Previous models that only included stiffness-mediated behaviors only turn fibrotic without being able to heal (Wellman et al., 2018). Similarly, our single-element models did not show any healing response to injury when strain sensitivity was removed (Supplementary Figure S1). Fibrosis also did not occur when the stiffness sensitivity was removed (Supplementary Figure S2). Previous self-healing models have been based on stiffness (Suki et al., 2020), however, the mechanism of healing based on stiffness is not supported by the positive feedback between stiffness and ECM deposition observed in fibroblasts (Liu et al., 2010; Moore and Herzog, 2013; Lin et al., 2017). These observations motivated the rationale that the self-healing component of the current model is caused by stretch, and the fibrotic component of the current model is caused by stiffness.

### Diverging network responses

The lungs and other organs in general, are able to sustain minor injuries and heal from them, but once fibrosis begins in diseases such as IPF, the tissue is unable to return to a healing state. In terms of feedback systems, these represent stable and unstable responses, respectively. There are two criteria we consider for what determines the outcome of an injury. The first is predisposition towards IPF, which can be caused by factors such as age, genetics, and smoking habits (American Thoracic Society, 2000), which is accounted for in Eq. 7 as the stiffness weight, $w_{2}$ . The effect of this sensitivity is shown in Figure 3, where for the same injury, a diverse set of responses is observed. The second criterion is the severity of the injury. While we impose different injury states onto the networks, the severity of an injury is not simple to quantify. Consider two injuries, one where 20% of the lungs is stiffened by 5x (Figure 2), and another where 10% of the lungs is stiffened by 10x (Figure 3). It is difficult to predict which injury will be worse, and if the results of similar injuries will be consistent.

To evaluate these diverse behaviors, we developed a fibrotic threshold, which accounts for both predisposition and environmental factors to determine if a system will become fibrotic. Thresholds such as this have been observed for differentiation into fibroblasts (Hinz, 2009). In this model, this threshold is defined as where the stable system becomes an unstable system and occurs when the stiffness response in Eq. 7 overpowers the strain response; that is to say, for an increase in *A*, the change in activation will increase, causing a positive-feedback response. In the analysis of the difference equations, this threshold is a single value, which depends on the stiffness sensitivity. In the first column of Figure 1, we observe that the threshold for fibrosis lowers as sensitivity increases, shown by the orange arrows, indicating that for the same injury, a more sensitive subject would develop fibrosis.

In the network model, however, springs interact such that the fibrotic threshold is a range of values, due to non-uniform strain. This means that a network with many springs with a stiffness below the threshold may appear to be fibrotic and much stiffer than a healthy lung, but will be able to heal, as seen in ARDS and PCPF (Herridge et al., 2011; Lorx et al., 2022). This also means that a network with few springs injured beyond the fibrotic threshold may be enough of a spark for the positive-feedback response. In Figure 2, 20% of springs were stiffened by 5x, and both healing and fibrotic responses can occur in the same tissue as a result of network effects. This diverging response cannot be predicted from just the injury or the predisposition; it necessitates a structural analysis of the network. Figure 2 demonstrates this fibrotic threshold as the boundary between the green self-healing response and the pink fibrotic region. Even though the injuries were all below the predicted threshold from the difference equations, shown where the black diagonal line crosses the pink-green boundary, the network effects are enough in some cases to push the network to becoming fibrotic. These results show that the network effects have an integral contribution to the outcome of an injury. Such behavior represents a microscopic bifurcation, and whether the fibrosis percolates to the macroscale depends on the local correlation structure of the injury. Hence, our model is able to recreate both healing and non-healing responses to fibrotic perturbations in ways similar to those observed in real diseases, providing insight into how the severity and spatial distribution of the initial injury as well as predisposition towards PF interact to either heal or not heal.

### Fibroblast to myofibroblast differentiation

In PF, there is an observed increase in the population of fibroblasts and myofibroblast populations (Moore and Herzog, 2013). There is debate over where myofibroblasts come from during PF (Moore and Herzog, 2013), so for this lumped-parameter model, we consider only myofibroblasts that occur from differentiation from fibroblasts. Previous models have typically maintained the number of agents for simplicity (Wellman et al., 2018; Suki et al., 2020; Hall et al., 2023); however, this model allows for the proliferation of agents. Additionally, the activation parameter allows us to make a distinction between fibroblasts and myofibroblasts based on their activation. As shown in Figure 6, we were able to capture a diverse set of differentiation responses based on the network’s sensitivity, ranging from partial differentiation (Figure 6A) to full differentiation (Figure 6E). We also analyzed the local activation accumulation in the networks (Figures 6B,D,F), which can be considered as an approximation of α-smooth muscle actin resulting from myofibroblast differentiation. Not only do we observe an increase in activation as fibrosis intensifies, we observe in Figure 6D that these highly activated agents are localized to fibrotic regions.

### Structural heterogeneity

One of the hallmarks of PF is the diversity of structures observed in the tissue, namely, honeycombing, fibrotic patches and consolidated tissue (Tanabe et al., 2020). In Figures 3, 4, we observe these structures in both networks and images of human tissue. When the network images are compared to the histological images in Figure 4, the similarities are obvious. Models with a high $w_{2}$ , such as 2.5 and 3, show consolidation and dense patches, and mid-sensitivity models, such as the $w_{2}$ = 2, show clear honeycombing structures. These structures were not directly coded into the model, but are emergent properties arising from the interactions of the agents with the mechanical model. While the broad behavior of the overall networks can be predicted from the difference equations, the structures that arise from these networks cannot be predicted, showing the necessity of utilizing structural networks in evaluating disease progression. The similarities between the networks and tissue images also indicate that the rules that define this model may closely match those that govern remodeling in real tissue.

During the progression of fibrosis in the network models with varying $w_{2}$ values, it was not immediately clear if all the networks had the same structures, just at different stages of progression. That is to say, would Figure 3I eventually progress into Figure 3P Zero-stiffness springs in the network have been digested such that *A* is reduced to zero, and this can be considered as ruptured springs. This is permanent remodeling and cannot be healed, as ruptured alveolar walls cannot be remade. The number of these zero-stiffness springs is therefore an indication of irreversible structural change and structural difference within the network. Upon analysis of the number of ruptured springs within the network, we found that the honeycomb structure had more ruptured springs than the dense fibrotic structure (Figure 5D). Because these springs cannot be remade, this shows that these networks are clearly structurally independent. The mechanism by which these structures arise is interesting and novel.

In models with a fibrotic response, injuries were initially individual patches randomly distributed through the tissue, as shown in Figures 2, 3. As the disease progresses, fibrotic bridges emerge between these patches forming larger fibrotic clusters. This behavior can be seen in the Figure 3, $w_{2}=$ 1.5 and 2 rows. We posit that this is caused by two mechanisms: (1) the lasting memory of highly-activated agents migrating away from fibrotic patches and (2) over-stretching the tissue between fibrotic patches, increasing activity and leading to over-deposition until the stiffness crosses into the fibrotic region. In the case of high stiffness sensitivity, this behavior continues until the whole region becomes fibrotic. However, in the moderate stiffness weight networks, once these bridges have formed, the characteristic rings seen in honeycombing form, causing that region of tissue to contract. This creates a region within the fibrotic ring that has both low stiffness and low strain, causing low activation and digestion of the tissue, leading to the rupture of the springs in that region, and creating honeycombing (Figures 3G–I). This is an important result because it suggests that remodeling is not only caused by over-stretching and breakage, which was not included in this model, but that pathological remodeling can be caused by the complex structural interactions of fibroblasts and the ECM. Interestingly, the percentage of broken springs does not exhibit a monotonic relationship with $w_{2}$ (Figures 5C,D); there is little breakage when the propensity for fibrosis is low ( $w_{2}$ is small), but breakage also decreases as $w_{2}$ increases above a certain level. Presumably because as weights increase there is an increase in wall breakage, then as the fibrotic response becomes stronger, the percent broken springs decreases. This appears to be in agreement with the honeycombing and dense fibrosis observed in Figure 3. Thus, the evolution of IPF tissue structure is a clear manifestation of network phenomena that cannot be understood by studying static cells in isolation.

This fibrotic bridging is similar in behavior to invasion percolation. While 10% of the springs in the network are stiffened by 10x, the initial stiffness of all networks is still near the initial value (Figure 5A), and the final progression of low $w_{2}$ networks show that the fibrotic regions are separated, and that the pathological remodeling has not percolated the network (Figure 3C). In the high $w_{2}$ networks, the progression leads to network percolation that leads to increased stiffness, and network configuration that results in the aforementioned pathological structures. This percolation also is evident in the contraction of the networks. Novel to the current network model is its ability to contract in response to disease progression. Previous models have had fixed boundaries that have held the network to a specific area or volume (Wellman et al., 2018; Suki et al., 2020). The third column in Figure 3 shows heterogeneity in final network area that is dependent on the stiffness sensitivity of the model, with the most contracted networks being percolated by fibrotic tissue, such as the dense fibrotic patch in Figure 3P. These results are consistent with the increased stiffness of these networks observed in Figure 5A and the difference equations’ behaviors in Figure 1. Further investigation of this percolation behavior is therefore warranted.

### Limitations

This model has a number of limitations that are important to address. First, this model approximates tissue as a set of linear springs with a constant Young’s modulus. In reality, tissue shows characteristic nonlinearity due to recruitment, and the Young’s modulus changes as disease progresses (Marchioni et al., 2021). Both of these characteristics add computational complexity to the simulations. In this context, simplification of constant Young’s modulus and use of linear springs are common assumptions in previous and current spring-network models. Furthermore, spring networks are typically two-dimensional and do not include airways for the same reasons listed above. To further limit computational complexity, the static strain of the network was used as an approximation of the cyclic stretch. For agent-based models, it is difficult to translate biological mechanisms into discrete values that can be applied via agents, which is why lumped-parameter models have been utilized to recreate broad behavior. The current model is no exception, where rather than the precise processes of digestion and deposition, we consider broad characteristic behaviors. The rate at which the agents migrate was determined by discrete steps across springs, rather than a specific speed or distance to cross in a given time. The median time from diagnosis to death for IPF is ∼3 years (Nathan et al., 2011) whereas our model has an approximate time of ∼5 months, however we consider this model, given the computational limitations, to be comparable to the time course of physiological disease progression. Furthermore, we did not consider the interactions of agents with the other fibrosis relevant cell types such as inflammatory cells, epithelial cells and endothelial cells. This simplification is also consistent with previous models that only considered one agent type. Despite these limitations, the current model appears robust and is able to recapitulate complex behaviors comparable to those seen physiologically.

## Conclusion

In this work, we developed a mechanosensitive agent-based model and spring network model hybrid that was capable of both fibrosis and healing based on different injury conditions. The inclusion of fibroblast-like-agents that were sensitive to both stiffness and stretch recapitulated these behaviors, and the application onto a network model led to the development of heterogeneous structures typical of PF. These results suggest that the interactive mechanical signals present in the lungs, i.e., stiffness and stretch, play a key role in both homeostatic maintenance and disease progression.

## Data Availability

The original contributions presented in the study are included in the article/Supplementary Material, and further inquiries can be directed to the corresponding author.
