# Supplementary material for: Elucidating the interaction between stretch and stiffness using an agent-based spring network model of progressive pulmonary fibrosis
Source: Front Netw Physiol. 2024 May 22;4:1396383. doi: 10.3389/fnetp.2024.1396383 (PMC11150662; doi:10.3389/fnetp.2024.1396383)
Supplement: Supplementary file 1 [file DataSheet1.pdf]

SUPPLEMENT

Agent Memory

A simplified version of Eq. 9 in the main text is

$$\Delta a = r[Z - a_i] \tag{S.1}$$

so

$$a_{i+1} = a_i + \Delta a = a_i + r[Z - a_i] \tag{S.2}$$

where $Z = w_1 a_{\epsilon,j} + w_2 a_{K,j} - c$ is the combined contributions from the strain and stiffness responses, a_i is the activation at the current iteration, and a_{i+1} is the activation at the next iteration. If $r = 0$, then

$$\Delta a = 0 \tag{S.3}$$

and

$$a_{i+1} = a_i. \tag{S.4}$$

Under these conditions, agents cannot react to their environment, so their behaviors are determined only by initial conditions.

At the other extreme, if $r = 1$, then

$$a_{i+1} = a_i + Z - a_i = Z. \tag{S.5}$$

In this case, a_{i+1} does not depend on a_i, so agents react only to their current environment without any memory of prior exposures. When $0 < r < 1$, agent behavior is influenced to some extent by the environment.

Dynamics of Agent Population

We consider a network of s springs with n nodes. To account for the steady migration of agents into the network from external pools, we let a new agent appear at each node in the network at each time step with probability p_1, so $p_1 n/s$ is the number of new agents per time step.

The probability that an agent will die at each iteration is p_2, so $p_2 D$ is the number of agents dying at each time step where D is current density of agents on the network.

The probability that an activated agent will divide into two independent agents at each iteration is p_3. This probability increases in proportion to the level of activation, a. When a is negative, the density of agents decreases.

The change in D at each time step is the sum of the three contributions listed above:

$$\Delta D = \frac{p_1 n}{s} - p_2 D + p_3 a D = \frac{p_1 n}{s} + [p_3 a - p_2]D. \tag{S.6}$$

Equation S.6 has two steady state solutions, $a = 0$ and $a = w_2 - 0.5$, which pertain when $\Delta D = 0$. Under these conditions, using the initial density D_0, Eq. S.6 rearranges to

$$D_0 = \frac{p_1 n}{s[p_2 - p_3 a]}. \tag{S.7}$$

When the tissue is healthy and not activated, this equation rearranges to

$$\frac{p_1 n}{s D_0} = p_2. \tag{S.8}$$

The tissue can only sustain a finite maximum agent density, D_{max}, which occurs when $a = w_2 - 0.5$. If we define the quantity

$$X = \frac{D_{max}}{D_0} \tag{S.9}$$

then Eq. S.7 becomes

$$D_0 X = \frac{p_1 n}{s[p_2 - \ p_3(w_2 - 0.5)\ ]}. \tag{S.10}$$

Solving for p_3 gives

$$p_3 = \frac{p_2\left(1 - \frac{1}{X}\right)}{(w_2 - 0.5)}. \tag{S.11}$$

Network Timescale

We ran the networks for 2000 iterations, which was enough to see both self-healing and saturation behaviors in both network models. Based on Tschumperlin (2013), fibroblasts travel at around 1μm/minute on soft substrates. In a six-sided 2-D alveolus of 200μm diameter, each spring in the hexagon is approximately 100 μm long. This means that each simulation is approximately 200,000 minutes, or approximately 4.6 months. That is,

$$2000\ iterations * \frac{1\ spring}{1\ iteration} * \frac{\sim 100\ \mu m}{1\ spring} * \frac{1\ minute}{1 \mu m} * \frac{1\ month}{43200\ minutes} = 4.6\ months. \tag{S.12}$$

This estimate does not take agent stalling into account, so it represents a lower bound.

Difference Equation Behaviors with No Strain and No Stiffness Component

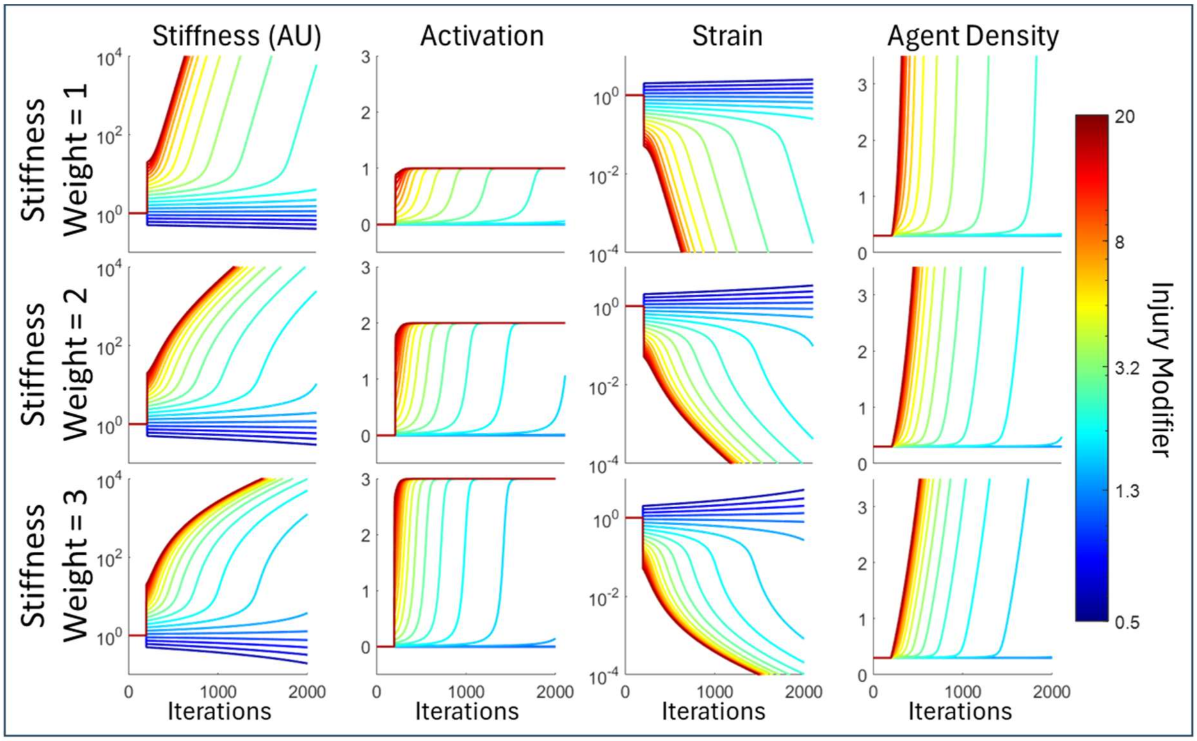

Supplemental Figure 1: Difference Equations with $w_1 = 0$. In this figure, the difference equations only have activation contributions from the stiffness of the underlying springs. Each of these networks appear to be unstable and does not show self-healing behaviors for any injury or for any of the values of w_2.

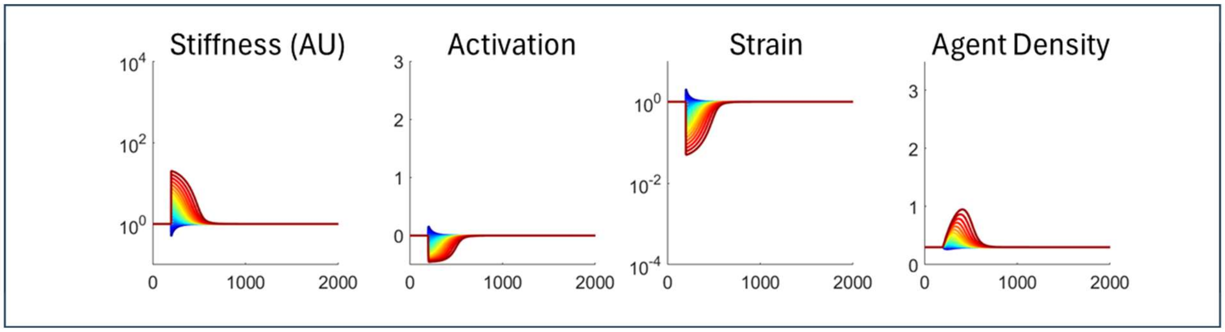

Supplemental Figure 2: Difference equations with $w_2 = 0$. In this figure, the difference equations only have activation contributions from the strain of the underlying springs. Here, no matter the injury, the system always heals.

<u>Comparison of Hexagonal Network and Non-uniform Network</u>

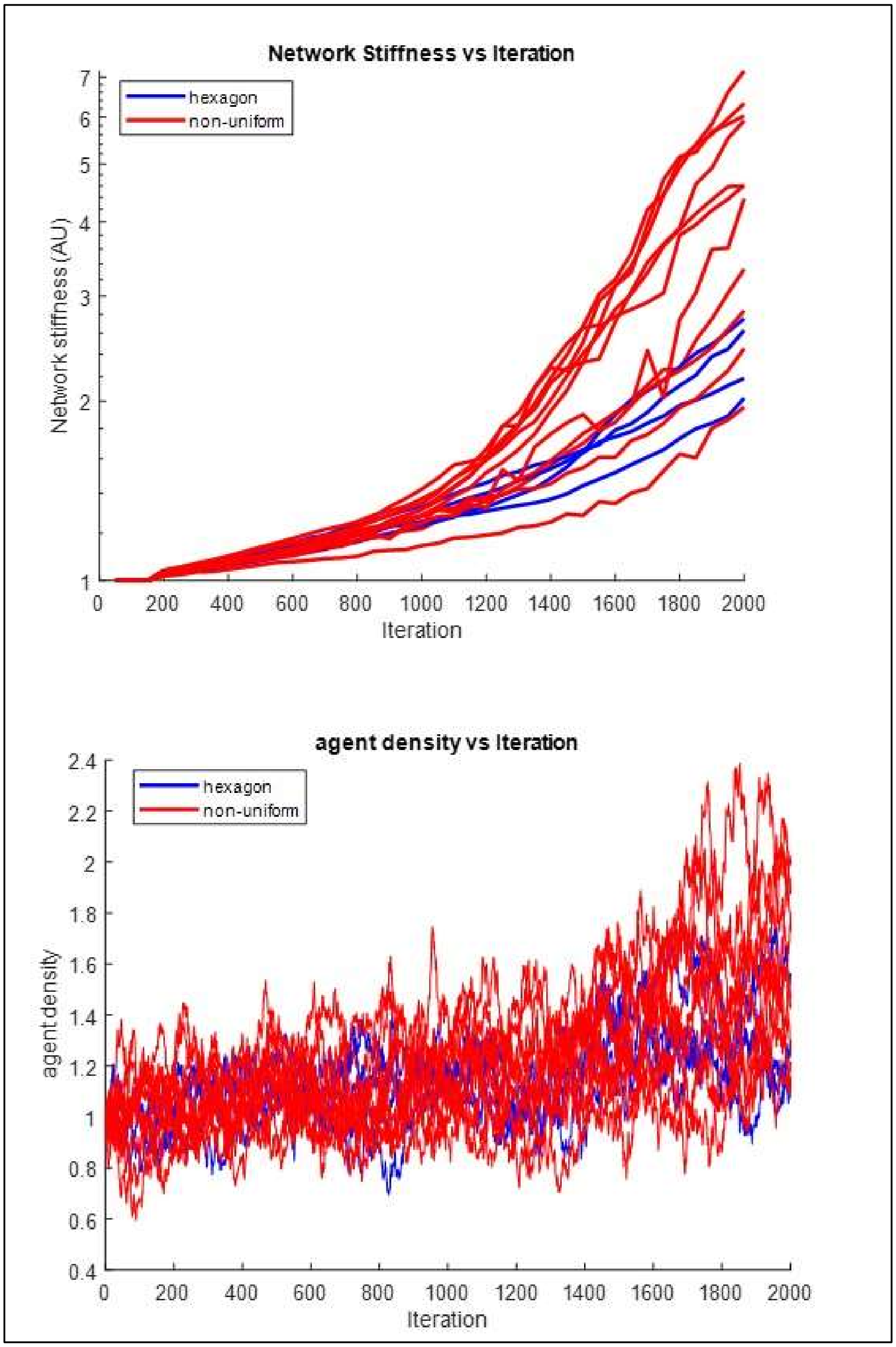

Supplemental Figure 3: Non-uniform network vs. hexagonal network behavior. A hexagonal network was created for comparison against the non-uniform networks. The results do not show any dramatic changes in the network stiffness or agent density behaviors.

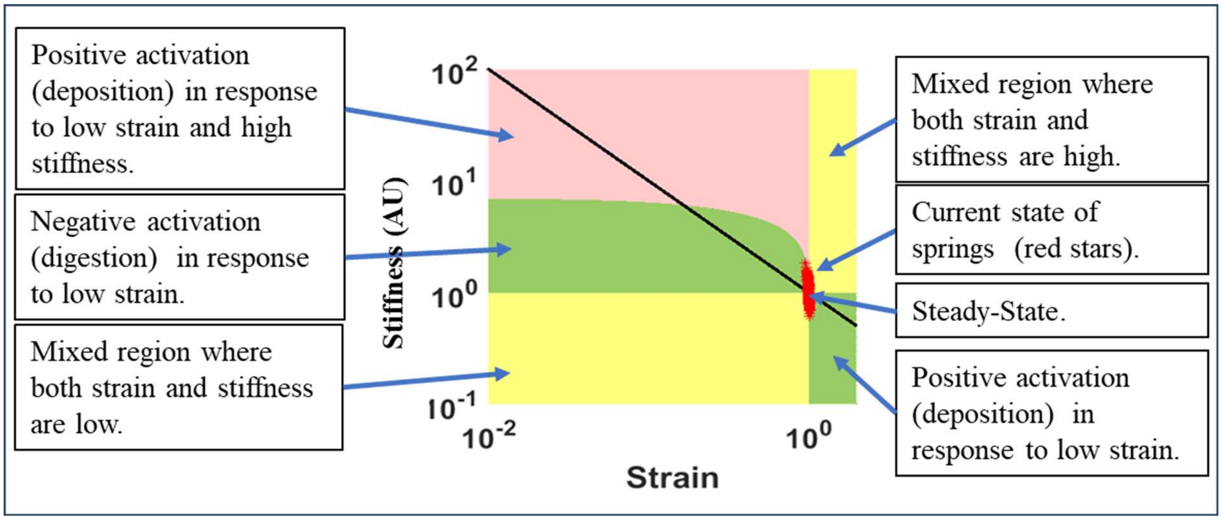

Supplemental Figure 4: Layout of a network phase diagram. The phases of this diagram, indicated by colors green, yellow, and pink, correspond to the activation of an agent in response to the conditions of the spring it is currently on according to Eqs. 3-5 in the main text. For the green regions, the activation tends towards returning to homeostasis, e.g. the lower right region, where strain is too high, has a positive activation to return to homeostasis. The upper left green region, where strain is too low, has a negative activation to return to homeostasis. The pink region corresponds to a positive activation in response to low strain and high stiffness, and the transition from green to pink occurs where the stiffness of the spring overwhelms the strain response, resulting in positive feedback. The yellow regions represent where stiffness and strain are low (lower left), and where the stiffness and strain are high (upper right). These regions are caused by the structure of the network imposing non-homeostatic conditions on the springs. In testing, these regions showed both healing and pathological responses, as shown in Fig. 2 D-I.
